# Single-Center Experience with the HeartMate 3 Assist Device in Pediatric Patients with Cardiomyopathy

**DOI:** 10.3390/medicina62010064

**Published:** 2025-12-28

**Authors:** Michal Hulman, Panagiotis Artemiou, Danielle Stuschkova, Stefan Durdik, Matej Nosal, Zuzana Hrubsova, Lubica Kovacikova, Pavol Kunovsky, Martin Zahorec, Matus Kovac, Ivo Gasparovic

**Affiliations:** 1 Department of Cardiac Surgery, National Institute of Cardiovascular Diseases, Medical Faculty, Faculty of Medicine, Comenius University, 84215 Bratislava, Slovakia; michal.hulman@nusch.sk (M.H.); daniellepaulina.stuschkova@nusch.sk (D.S.); ivo.gasparovic@nusch.sk (I.G.); 2 Faculty of Medicine, Comenius University, 84215 Bratislava, Slovakia; stefan.durdik@ousa.sk; 3 Department of Pediatric Cardiac Surgery, National Institute of Cardiovascular Diseases, Faculty of Medicine, Slovak Medical University, 84215 Bratislava, Slovakia; matej.nosal@nusch.sk; 4 Department of Pediatric Cardiology, National Institute of Cardiovascular Diseases, Faculty of Medicine, Comenius University, 84215 Bratislava, Slovakia; zuzana.hrubsova@nusch.sk (Z.H.); lubica.kovacikova@nusch.sk (L.K.); pavol.kunovsky@nusch.sk (P.K.); martin.zahorec@nusch.sk (M.Z.); matus.kovac@nusch.sk (M.K.)

**Keywords:** mechanical assist device, HeartMate 3 assist device, outcomes

## Abstract

*Background and Objectives*: The use of the HeartMate 3 (HM3) ventricular assist device (VAD) as a bridge to transplant is rising notably in pediatric patients with end-stage heart disease. This study reports a single-centre experience with the HM3 device in pediatric patients. *Materials and Methods*: We conducted a retrospective clinical data review (including procedural, perioperative and follow-up parameters) of pediatric patients (*n* = 5, aged 10–16 years) supported with HM3 VAD at our institution between January 2022 and October 2025. *Results*: During this period, five pediatric patients (median age of 13 years [range 10–16, IQR 10–14.5]; median weight of 41.5 kg [24–75 IQR 32–62]) underwent HM 3 implantation. The Pedimacs profiles ranged from 1 to 2. Two patients with Pedimacs profile 1 received a temporary left ventricular assist device preoperatively for hemodynamic stabilization. The median intensive care unit stay was 47 days (range 21–54, IQR 28–50.5). Right ventricular dysfunction occurred in four patients and was managed conservatively in three, while one required intraoperative implantation of a temporary CentriMag right ventricular assist device. Two patients remain on device support, while three patients were successfully bridged to heart transplantation. No mortality was observed, and none of the patients experienced pump-related complications. *Conclusions*: The use of the HeartMate 3 is a safe and effective treatment strategy for successful bridging to heart transplantation. Right ventricular dysfunction is a common and clinically significant complication, but it can be effectively managed with appropriate measures.

## 1. Introduction

Dilated cardiomyopathy is frequently diagnosed at an advanced stage in pediatric patients, with a reported annual incidence ranging from 0.57 to 6.95 cases per 100,000 patients [1,2]. For patients with end-stage heart failure due to dilated cardiomyopathy, heart transplantation represents the optimal treatment option. A well-established bridging therapy for pediatric patients awaiting transplantation is the mechanical circulatory support using the Berlin Heart EXCOR system (Berlin Heart, Berlin, Germany) [3]. According to the recent analysis by the United Network for Organ Sharing (UNOS), it was the most frequently used ventricular assist device (VAD), with 52.8% [4].

To enhance mobility and facilitate reintegration into daily life, efforts have been directed towards the development of intracorporeal devices. Widespread implementation of these devices in the pediatric population has been constrained by two principal factors: the anatomic challenge posed by their considerable size in smaller patients and a higher risk of thromboembolic events and bleeding complications [5,6]. As left ventricular assist devices (LVADs) continue to improve in terms of miniaturization and hemocompatibility, their use in pediatric patients is expected to increase.

One such device designed to partially overcome these limitations is the HeartMate 3 (HM3) device (Abbott Corp., Lake County, IL, USA) by utilizing a fully magnetically levitated continuous centrifugal-flow mechanism. Nevertheless, the size mismatch between the device and smaller pediatric patients remains a significant challenge, resulting in a limited number of studies reporting experience with the HM3 in this population [7].

In order to evaluate the safety and efficacy of the HeartMate 3 in a pediatric cohort, we performed a retrospective review of patients with end-stage heart failure secondary to dilated cardiomyopathy at our institution. The novelty of our cohort lies in the inclusion of a pediatric patient at the lower extreme of the size spectrum, where HM3 implantation is the most technically demanding. Moreover, two patients were transitioned from a temporary to a durable mechanical circulatory support.

## 2. Materials and Methods

We conducted a retrospective clinical data review (including procedural, perioperative and follow-up parameters) of pediatric patients (*n* = 5, aged 10–16 years) who received HM3 support for end-stage heart failure secondary to dilated cardiomyopathy at our institution between January 2022 and October 2025. Written informed consent from the patients’ legal guardians was obtained for inclusion in this study. The study was approved by the institutional review board (protocol code 15_2025 and date of 12 December 2025).

All patients were evaluated by a multidisciplinary team and, following a comprehensive assessment, were deemed suitable candidates for HM3 implantation. Pedimacs profiles were also assessed [8]. The criteria for HM3 implantation were as follows:

1. New York Heart Association (NYHA) class IV;
2. Pedimacs profiles 1–2;
3. heart failure symptoms refractory to guideline-directed medical therapy;
4. left ventricular ejection fraction < 25%;
5. continuous intravenous inotropic support due to symptomatic hypotension, declining renal function, or worsening pulmonary congestion.

Patients classified as Pedimacs profile 1 prior to HM3 implantation received short-term mechanical circulatory support for hemodynamic stabilization.

The decision to implant an LVAD is based on echocardiographic and clinical parameters. Echocardiographic parameters for right ventricular function assessment included fractional area change (FAC) > 20%, free wall strain > 9.6%, and tricuspid annular plane systolic excursion (TAPSE) > 8 mm.

Right ventricular dysfunction after the LVAD implantation was defined as the presence of clinical (persistent low LVAD flow, hypotension, escalating inotropic therapy, worsening end-organ dysfunction-rising lactate, hepatic congestion, and renal impairment), echocardiographic (right ventricular dilatation and systolic dysfunction, FAC < 35%, TAPSE < 10 mm, interventricular septal shift to the left, and moderate or severe tricuspid regurgitation), and/or hemodynamic (elevated central venous pressure > 15 mmHg, increased central venous pressure/pulmonary artery wedge pressure ratio > 0.63, and cardiac index < 2.2 L/min/m^2^ despite adequate LVAD flow) evidence of inadequate right ventricular performance. Indications for temporary right ventricular assist device (RVAD) were inability to wean from cardiopulmonary bypass due to right ventricular dysfunction and dilatation, sustained CVP ≥ 18–20 mmHg with low LVAD flows, LVAD suction events, requirement for high-dose or escalating inotropic/vasopressor support beyond 24–48 h, progressive end-organ dysfunction, and echocardiographic evidence of severe right ventricular dysfunction with interventricular septal distortion unresponsive to medical therapy.

Management of right ventricular dysfunction followed a stepwise approach with escalation based on response to therapy: optimization of preload and afterload (volume management, reduction of pulmonary vascular resistance and correction of hypoxia, hypercapnia and acidosis, pharmacologic support with inotropes and vasopressors) and mechanical and ventilatory strategies (optimization of LVAD speed, ventilatory management minimizing mean airway pressure and excessive positive end-expiratory pressure). Temporary RVAD was considered in patients with refractory right ventricular dysfunction despite optimal medical and ventilatory management.

The HM3 is a fully magnetically levitated LVAD that provides intrinsic pulsatility with minimal shear stress, thereby reducing blood stasis and the activation of thrombogenic components [9]. A median sternotomy was performed, and cardiopulmonary bypass was established with aortic and right atrial cannulation. All device implantations were performed on a beating heart without the use of cardioplegia. The inflow cannula was inserted through the left ventricular apex, and the outflow graft was anastomosed to the proximal ascending aorta using a side-biting clamp.

Preoperatively, the feasibility of device placement and its optimal positioning were evaluated using multiple imaging modalities. Two patients underwent magnetic resonance imaging (MRI), two patients underwent computed tomography (CT) imaging, and Patient 5 did not undergo any additional preoperative imaging examination based on heart team consensus.

Postoperatively, all patients required intravenous inotropes (primarily adrenaline, noradrenaline, and milrinone), which were gradually tapered according to clinical status. Anticoagulation therapy was initiated with intravenous heparin (10–20 IU/kg/h), followed by warfarin therapy (target international normalized ratio [INR] 2.0–2.5), with the exception of Patient 1 who received a combination of warfarin and aspirin (100 mg/day). The study endpoints included complications, mortality, and clinical outcomes during HM3 support. The adverse events assessed comprised postoperative bleeding or cardiac tamponade requiring surgical re-exploration; pleural effusions; arrhythmias (atrial fibrillation); infections, including sternal wound infections as well as respiratory and urinary tract infections; and acute renal failure requiring dialysis and respiratory impairment (escalation of respiratory support, prolonged ventilation, hypoxemia, hypercapnia, and radiologic and clinical findings).

Follow-up examinations were scheduled in accordance with institutional protocols (early outpatient follow-up: 2 weeks and 1 month after discharge; intermediate follow-up: every 2–3 months during ongoing HM 3 support; long-term follow-up: every 3–6 months for prolonged support).

Categorical variables were expressed as numbers and percentages, and continuous variables were expressed as medians with interquartile ranges (IQRs).

## 3. Results

During the study period, five patients underwent HM3 implantation, with a median age of 13 years (range 10–16, IQR 10–14.5), median body surface area of 1.32 m^2^ (range 0.96–1.97, IQR 1.13–1.73), and median weight of kg 41.5 (24–75, IQR 32–62). The Pedimacs profiles of the patients ranged from 1 to 2. Preoperatively, only one patient exhibited life-threatening arrhythmia (ventricular tachycardia). Two patients with Pedimacs profile 1 received temporary LVAD support for hemodynamic stabilization prior to HM3 implantation, using CentriMag (Abbott Corp., USA) and Impella CP (Abbott Corp., USA) for 2 and 7 days, respectively. Table 1 outlines the preprocedural demographic and clinical characteristics of the study cohort.

The median cardiopulmonary bypass time was 67 min (range 52–152, IQR 54–120.5). One patient required intraoperative implantation of a temporary RVAD using the CentriMag system (Abbott Corp., USA), with the arterial cannula inserted into the pulmonary artery through an elephant trunk graft exteriorized subxiphoidally and the venous cannula placed percutaneously via the femoral vein. The RVAD was explanted after right ventricular recovery on postoperative day (POD) 7. The sternum was closed intraoperatively in all patients with the exception of Patient 4, who underwent delayed sternal closure.

The median intensive care unit (ICU) stay was 47 days (range 21–54, IQR 28–50.5), and the median hospital stay was 50 days (range 28–63, IQR 36–62.5). Patients received inotropic support for a median of 12 days (range 8–25, IQR 9–23.5). The inotropes administered included different combinations of adrenaline, noradrenaline, milrinone, levosimendan, and dobutamine according to patient clinical status. All patients received pulmonary hypertension therapy with inhaled nitric oxide (iNO) and sildenafil.

Right ventricular dysfunction occurred in four patients and was managed conservatively in three, while one required intraoperative implantation of a temporary CentriMag RVAD. Endomyocardial biopsy was performed only in Patient 5, revealing an old organized subendocardial infarction with an organizing mural thrombus at the site of necrosis and mild endocardial fibrosis. Intraoperative and postoperative data are presented in Table 2.

The median follow-up duration was 19 months (range 1–46, IQR 5.5–35), and the median duration of device support was 7.5 months (range 1–24, IQR 2–17). Two patients remain on device support at 10 and 24 months, while three patients were successfully bridged to heart transplantation after 1, 3, and 7.5 months, respectively. No mortality was observed during the study period. All patients were discharged home after HM3 implantation; three resumed regular school attendance, one continued with home-based education, and one underwent heart transplantation one week after discharge. Furthermore, none of the patients experienced complications such as pump thrombosis, stroke, seizures, neurological deficits, or device-related infection while on HM3 support. The follow-up data is summarized in Table 2.

Finally, in our cohort, two patients (patients 1 and 5) presented with Pedimacs profile 1; therefore, temporary VAD support was initiated to achieve hemodynamic stabilization. Patient 1 received preoperative support for 2 days with the CentriMag assist device, whereas Patient 5 underwent percutaneous implantation of an Impella CP device (Abbott, USA), providing circulatory support for 7 days. Temporary VAD support resulted in hemodynamic stabilization and reversal of end-organ hypoperfusion in both patients, as evidenced by the normalization of lactate levels. In patient 1, creatinine, total bilirubin, and INR declined from 50 µmol/L to 41 µmol/L, from 21.5 µmol/L to 18.5 µmol/L, and from 2.40 to 1.35, respectively. Similarly, in patient 5, creatinine decreased from 87 µmol/L to 32 µmol/L, INR from 1.52 to 1.19, and total bilirubin from 22.6 µmol/L to 9.7 µmol/L. After achieving hemodynamic stabilization, the decision to transition both patients from temporary to durable LVAD support was made.

Four patients developed right ventricular dysfunction immediately after LVAD implantation. In three patients (patients 1, 2, and 4), the condition was managed with inotropic therapy, whereas the remaining patient (patient 5) required intraoperative implantation of a temporary RVAD, which was successfully explanted 7 days later.

## 4. Discussion

Ventricular assist devices play a significant role in the treatment of end-stage heart failure, with their use steadily increasing. However, experience in the pediatric population remains limited due to the lack of smaller-sized devices and the wide spectrum of underlying pathologies encountered. The Berlin Heart EXCOR (Berlin Heart, Berlin, Germany) and the HeartWare HVAD (Medtronic Inc., Framingham, MA, USA) were the main devices used in this population until 2021, when the HVAD was withdrawn from the market. Despite their smaller profile, both devices have been associated with higher rates of stroke and thrombus formation [10].

The HM3 is a frictionless, magnetically levitated centrifugal pump designed to generate an intrinsic artificial pulse, thereby reducing blood stasis. In the MOMENTUM 3 trial, hemocompatibility-related adverse events at two years showed a near elimination of pump thrombosis, a 50% reduction in stroke rates, and a marked decrease in both surgical and non-surgical bleeding events compared with the HeartMate II axial-flow pump [11].

Although heart transplantation remains the gold standard for treating end-stage heart failure, the availability of devices such as the HM3 in the pediatric armamentarium offers many advantages. The major limitation of HM3 implantation in pediatric patients is the restricted intrathoracic space and the potential risk of compromised pump positioning during chest closure [12].

The Advanced Cardiac Therapies Improving Outcomes Network (ACTION) reported a series of 35 pediatric patients who underwent HM3 implantation. Similar to our cohort, the majority were classified as Pedimacs profile 2, and 37% received some form of mechanical circulatory support (extracorporeal membrane oxygenation or paracorporeal/percutaneous continuous-flow devices) prior to HM3 implantation. In our study, one patient was preoperatively supported with a paracorporeal continuous-flow device and another with a microaxial flow pump. Right ventricular dysfunction following HM3 implantation was more frequent in our cohort. Notably, none of our patients experienced pump thrombosis, stroke, seizures, neurological deficits, or infection during device support, in contrast to the ACTION registry, where these complications were more prevalent [7]. In our study, three patients were successfully bridged to heart transplantation, and two remain on device support. In comparison, in the ACTION registry, 61.2% were bridged to transplant, 22.9% remained on device support, 2.4% achieved recovery with explantation, and 7.6% died while on support.

The increasing use of the HM3 in pediatric patients is further reflected in the fourth Pediatric EUROMACS (Pedi-EUROMACS) report [3], which documented an increase in HM3 use from 4.1% to 5.4% (as of August 2023, 32 patients). The median age and body size of the patients were similar to those in our cohort. The smallest child who received an HM3 was 10 years old with a body surface area (BSA) of 0.91 m^2^, comparable to the smallest patient in our study (10 years old, 24 kg, BSA 0.91 m^2^). As in our series, cardiomyopathy was the most common underlying diagnosis. Pedimacs profiles 1/2 and continuous-flow devices were independent predictors of mortality, whereas no deaths occurred in our cohort despite the presence of similar Pedimacs profiles.

Moreover, the use of the HM3 device in pediatric patients in recent years is also reflected in a recent UNOS analysis (1 January 2014, to 31 December 2023), in which 5.8% of patients received the HM3 as a bridge-to-transplant strategy [4].

The sixth Pedimacs report [13] provided broader categories of VADs, noting that implantable continuous-flow devices (including the HM3) accounted for 40% of implants, with cardiomyopathy being the leading indication. At six months post-implantation, a favorable outcome was achieved in 84% of patients. In our series, two patients underwent heart transplantation within six months, and a third was transplanted after 7.5 months of device support.

Beyond these registries, published evidence remains limited to case reports and small case series. Successful HM3 implantations in pediatric patients have been described by Bansal et al. [9], Azeka et al. [14], Muntean et al. [15], Haselmann et al. [16], and Zumwalt et al. [17] in a patient with muscular dystrophy. These reports collectively suggest that the HM3 represents a valuable and reliable bridge-to-transplant option in pediatric patients.

Trezzi et al. [18] reported a series of seven pediatric patients with a median duration of device support of 138 days, compared with a median of 10 months in our cohort. Similar to our findings, no patient experienced stroke, pump thrombosis, or infection, and two patients were successfully transplanted. Dogan et al. [19] described a series of 11 patients who developed early complications such as pleural effusions and tamponade, consistent with our experience. However, in their study, only one patient was bridged to transplantation, and one patient died during follow-up. In comparison to our series, three of five patients were transplanted, and no deaths occurred during device support.

Several concerns are associated with HM3 implantation in pediatric patients. The first relates to whether the pump can be safely accommodated within the limited thoracic cavity without compromising LVAD flow. In our experience, no such complications occurred, and all implantations were uneventful. To address this issue, preoperative CT–based virtual simulation has been used to assess the feasibility of device placement and determine optimal pump positioning. Furuta et al. [20] utilized CT virtual simulation to successfully implant a HM3 in a pediatric patient, and Ushijima et al. [21] also described a modified surgical technique facilitating safe implantation in small children.

A second concern is the potential for frequent device alarms despite optimized pump settings. In our cohort, the actual flow rates were consistently around or above 3 L/min, and frequent alarms were not encountered during the early postoperative period.

Right ventricular dysfunction is a common and clinically significant complication after LVAD implantation. Preoperative right ventricular impairment, a severe clinical profile (Pedimacs profiles 1–2), and urgent or emergency implantation may increase the likelihood of its occurrence. Conversely, thorough preoperative optimization, careful adjustment of LVAD function, the use of pulmonary vasodilatory and respiratory management strategies, and the timely escalation to mechanical right ventricular support have a protective effect on right ventricular performance [22,23]. Potential explanations for the occurrence of right heart dysfunction in our cohort include Pedimacs profiles 1 and 2, preexisting right ventricular impairment, and end-organ dysfunction. The HM3 devices were implanted in a timely manner, and patients presenting with Pedimacs profile 1 underwent temporary VAD implantation to achieve preoperative hemodynamic stabilization and reversal of end-organ hypoperfusion. Postoperatively, LVAD function was optimized, and pulmonary vasodilation therapy with inhaled nitric oxide and sildenafil was administered. Additionally, one patient required intraoperative escalation to mechanical right ventricular support due to refractory right ventricular dysfunction.

The limitations of our study include the small number of patients and its retrospective, single-center design. The interpretation of our conclusions regarding the safety and efficacy of the HM3 in pediatric patients is limited by the small sample size and therefore cannot be generalized beyond the context of our single-center experience.

## 5. Conclusions

Despite the small patient cohort, our single-center experience suggests that use of the HM3 in pediatric patients with end-stage heart failure is feasible and appears to be associated with acceptable short-term outcomes, including successful bridging to heart transplantation. No mortality was observed in this limited series; however, these findings should be interpreted cautiously given the sample size. Right ventricular dysfunction emerged as a frequent and clinically relevant complication in this population, underscoring its importance in perioperative management. In our experience, targeted preoperative assessment and tailored intraoperative and postoperative strategies were associated with effective management of right ventricular dysfunction.

## Figures and Tables

**Table 1:** Pre-implantation patients’ characteristics.

	Patient 1	Patient 2	Patient 3	Patient 4	Patient 5
**Age (years)**	10, female	16	13	13	10, female
**PAEDIMACS profile**	1	2	2	2	1
**Weight (kg)**	24	75	49	41.5	40
**Body surface area (m^2^)**	0.96	1.97	1.48	1.32	1.30
**Etiology**	Dilated cardiomyopathy	Hypertrophic cardiomyopathy with secondary dilation	Chemotherapy-induced dilated cardiomyopathy	Dilated cardiomyopathy	Dilated cardiomyopathy
**Arrhythmia**	-	AF, VT	-	-	-
**Device type/time of implantation** **Preoperative support**	HM3-01/2022 CentriMag tLVAD	HM3-10/2023	HM3-03/2024	HM3-12/2024	HM3-08/2025 Impella CP tLVAD
**Hemodynamic parameters**			-		-
CVP, mmHg	21	10		25	
PAWP, mmHg	23	21		22	
PAPi	2	3.2		3	
**Laboratory parameters**					
Total bilirubin, µmol/L	18.5	25.8	7.2	24.2	9.7
Creatinine, µmol/L	41	74	56	52	31
NTproBNP, ng/L	16,048	3382	5748	7528	5709
**Echocardiographic parameters**					
EF, %	25	25	25	12	10
LVEDD, mm	60	77.2	50.8	72	60
TAPSE, mm	17.5	21	11	17.5	11
FAC RV, %	19	56	24	28	21

AF = atrial fibrillation, VT = ventricular tachycardia, HM3 = HeartMate 3, tLVAD = temporary left ventricular assist device, CVP =central venous pressure, PAWP = pulmonary artery wedge pressure, PAPi = pulmonary artery pressure index, NTproBNP = N-terminal pro brain natriuretic peptide, EF = ejection fraction, LVEDD = left ventricular end-diastolic diameter, TAPSE = tricuspid annular plane systolic excursion, FAC = fractional area change, RV = right ventricle.

**Table 2:** Intraoperative, postoperative and follow-up data of the patients.

	Patient 1	Patient 2	Patient 3	Patient 4	Patient 5
**Cardiopulmonary bypass duration (min)**	67	52	56	89	152
**Device average RPM**	4600	5300	4600	4500	4700
**ICU stay (days)**	47	21	54	35	47
**Post-op iv inotropes, duration, days**	10	8	12	25	22
**Hospital stay (days)**	62	28	63	44	50
**Early postoperative complications**	Pleural effusion-drainage Pneumonia/empyema Atrial fibrillation Hypertension	Pneumonia Pleural effusion Atrial fibrillation Hypertension Sternal wound infection	Pleural effusion	Bleeding/tamponade Pleural effusion	Pleural effusion
**PHT treatment** **(iNO, sildenafil**)	+	+	+	+	+
**Right ventricular dysfunction**	+	+	−	+	tRVAD CentriMag
**Follow-up duration (months)**	46	24	19	10	1
**Outcome**	After 7.5 months on device support underwent HTx,-doing well	Still on device support 24 month, -awaiting HTx	After 3 months on device support, underwent HTx-doing well	Still on device support-10 months awaiting HTx	After 1 month on device support underwent HTx-doing well

RPM = rounds per minute, ICU = intensive care unit, PHT = pulmonary hypertension, NO = nitric oxide, tRVAD = temporary right ventricular assist device, HTx = heart transplant.

## Data Availability

The data underlying this article contain sensitive patient information and cannot be shared publicly due to institutional and ethical restrictions. De-identified data may be made available upon reasonable request to the corresponding author and with appropriate ethical approval.
